# Analysis of the mouse embryonic stem cell regulatory networks obtained by ChIP-chip and ChIP-PET

**DOI:** 10.1186/gb-2008-9-8-r126

**Published:** 2008-08-13

**Authors:** Divya Mathur, Timothy W Danford, Laurie A Boyer, Richard A Young, David K Gifford, Rudolf Jaenisch

**Affiliations:** 1 Department of Biology, Massachusetts Institute of Technology, Ames Street, Cambridge, MA 02139, USA; 2 Whitehead Institute for Biomedical Research, Cambridge Center, Cambridge, MA 02142, USA; 3 Computer Science and Artificial Intelligence Laboratory, Massachusetts Institute of Technology, Vassar Street, Cambridge, MA 02139, USA

## Abstract

**Background:**

Genome-wide approaches have begun to reveal the transcriptional networks responsible for pluripotency in embryonic stem (ES) cells. Chromatin Immunoprecipitation (ChIP) followed either by hybridization to a microarray platform (ChIP-chip) or by DNA sequencing (ChIP-PET), has identified binding targets of the ES cell transcription factors OCT4 and NANOG in humans and mice, respectively. These studies have provided an outline of the transcriptional framework involved in maintaining pluripotency. Recent evidence with comparing multiple technologies suggests that expanding these datasets using different platforms would be a useful resource for examining the mechanisms underlying pluripotency regulation.

**Results:**

We have now identified OCT4 and NANOG genomic targets in mouse ES cells by ChIP-chip and provided the means to compare these data with previously reported ChIP-PET results in mouse ES cells. We have mapped the sequences of OCT4 and NANOG binding events from each dataset to genomic coordinates, providing a valuable resource to facilitate a better understanding of the ES cell regulatory circuitry. Interestingly, although considerable differences are observed in OCT4 and NANOG occupancy as identified by each method, a substantial number of targets in both datasets are enriched for genes that have known roles in cell-fate specification and that are differentially expressed upon *Oct4 *or *Nanog *knockdown.

**Conclusion:**

This study suggests that each dataset is a partial representation of the overall ES cell regulatory circuitry, and through integrating binding data obtained by ChIP-chip and ChIP-PET, the methods presented here provide a useful means for integrating datasets obtained by different techniques in the future.

## Abstract 2

OCT4 and NANOG genomic targets were identified in mouse embryonic stem cells by ChIP-chip and were compared with previously reported ChIP-PET results.

## Background

Embryonic stem (ES) cells are derived from the inner cell mass of the embryo and possess the property of pluripotency, which is the ability to develop into any cell lineage of the organism [1-3]. The derivation of these cells has had significant impact on biomedical research and has important implications for regenerative medicine. Consequently, a detailed knowledge of the mechanisms governing pluripotency in ES cells is necessary to realize the potential of these cells. The homeodomain transcription factors OCT4 and NANOG are uniquely expressed in pluripotent cell types and have essential roles during development [4,5]. For instance, *Oct4 *knockout embryos and ES cells differentiate into trophectoderm, whereas over-expression of the gene leads to differentiation into primitive endoderm and mesoderm lineages [6,7]. Loss of *Nanog *in the early embryo and ES cells results in differentiation into primitive endoderm [8,9]. Conversely, NANOG over-expression obviates the need for the cytokine, leukemia inhibitory factor for ES cell self-renewal [8,9]. Collectively, these studies suggest that OCT4 and NANOG function in concert to regulate pluripotency in the early embryo, and similarly in ES cells to govern the transcriptional regulatory circuitry.

Recent genomic studies in ES cells have provided the foundation for understanding the genetic network that is the collective output of these pluripotency factors. Studies in both human and mouse ES cells have used chromatin immunoprecipitation (ChIP) combined with genome-wide technologies to uncover OCT4 and NANOG genomic binding events that may underlie transcriptional regulatory circuitries involved in maintaining a stem cell state [10-12]. Such investigations have shown that in both species, OCT4 and NANOG occupy a large number of transcriptionally active and silent genes, many of which are transcriptional regulators that have been implicated in lineage specification and cell fate determination. Moreover, a substantial overlap between the OCT4 and NANOG genomic targets exists within each dataset, suggesting that these two factors act in concert to regulate a common set of downstream pathways. This has been further substantiated by gene-expression studies following short hairpin RNA-mediated knockdown of *Oct4 *and *Nanog *[13].

ChIP coupled with a genome-wide DNA detection platform has been useful in studying protein-DNA interactions. The data obtained from these different platforms, however, are expected to exhibit variations due to the technical differences in the methods, as well as in data analysis. To date, ES cell binding data have been collected using ChIP-PET (paired-end ditags) [12] and ChIP-chip [11] for mouse ES cells and ChIP-chip for human ES cells [10]. However, comprehensive technological comparisons between ChIP-chip and ChIP-PET indicate that composite datasets that incorporate information from multiple platforms in a complementary fashion will be most useful in examining these networks in a comprehensive manner [14]. Such analysis is necessary since the binding data obtained from different platforms can vary due to the differences in sample processing for each method. In the study by Kim *et al*. [11], the authors provide a comparison between OCT4 and NANOG targets obtained from ChIP-chip and previously reported ChIP-PET data [12]. However, such overlap can vary dramatically depending on the thresholds used for determining bound regions by each experimental method. Since these thresholds are, to a large extent, arbitrary, it is important to examine how the binding data obtained by different platforms change under a wide range of threshold values.

To this end, we have employed ChIP-chip to identify the genomic binding targets of the pluripotency factors OCT4 and NANOG in mouse ES cells. Additionally, we have devised methods to examine these results along with previously published data for these factors using ChIP-PET under a wide range of binding thresholds [12]. All data have been re-mapped to the same version of the mouse genome, and provide a resource for studying this expanded transcriptional network obtained by integrating our ChIP-chip data and previously reported ChIP-PET results. Our analyses revealed substantially different sets of OCT4 and NANOG targets identified by each technique. However, a significant proportion of these targets included genes encoding transcription factors and other regulators of development in both datasets. Interestingly, many of the genes identified in both studies were differentially expressed upon *Oct4 *or *Nanog *knockdown in ES cells, suggesting that these targets were regulated by OCT4 and NANOG. Importantly, an examination of multiple data sources provided in this study has revealed a more comprehensive framework for understanding the mouse ES cell regulatory network.

## Results

### OCT4 and NANOG ChIP-chip binding data

**Figure 1:**
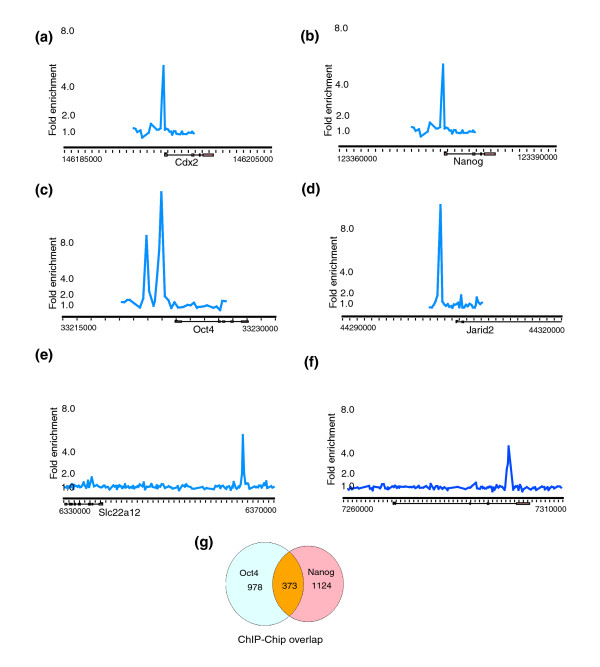
Illustrative examples of ChIP-enrichment ratios of **(a, b, e) **OCT4 and **(c, d, f) **NANOG bound regions obtained from ChIP-chip experiments with promoter arrays (a-d) and chromosome 19 arrays (e, f). The chromosomal position of the genes, as well as the genomic scale, is represented along the x-axis. The fold enrichment of the probes is shown on the y-axis. These enrichment ratios represent the medians of the per-pixel ratios scanned at each spot on the microarray. Exons and introns are represented by boxes and horizontal lines, respectively. The transcription start site and direction of transcription are denoted by arrows. **(g) **Venn diagram depicting the overlap between gene whose promoters were bound by both OCT4 and NANOG in ChIP-chip experiments (*p*-value < 0.001).

DNA sequences occupied by OCT4 and NANOG in mouse ES cells were identified in three independent biological replicates using ChIP as previously described [Arrayexpress: E-TABM-410] [10]. Samples were hybridized to microarrays that contained oligonucleotide probes that span the region -4 to +4 kb relative to the transcriptional start sites for 19,993 annotated mouse genes and 258 microRNAs [Arrayexpress: A-MEXP-957, Arrayexpress: A-MEXP-958]. Based on previously established criteria, bound regions were identified as peaks of ChIP-enriched DNA that span closely neighboring probes (Figure 1a-d) (Additional data files 1, 2, 15 and 16) [10]. Moreover, only those regions that were bound in all three replicates are represented in the final dataset. Using these stringent parameters, we identified 1,351 (6.8%) and 1,124 (5.6%) known protein-coding genes (Additional data file 5) and 22 (8.5%) and 23 (8.9%) microRNA genes (Additional data file 6) that are occupied by OCT4 and NANOG, respectively.

Several lines of evidence indicated that this ChIP-chip dataset is of high quality (see Additional data files 13 and 15 for error rate estimation by gene-specific PCR). First, in accordance with previous findings in both mouse and human ES cells [10,12], Gene Ontology (GO) analyses revealed that a significant number of promoters occupied by OCT4 and NANOG contained transcription factors and genes involved in developmental processes (Additional data file 7). Some of these genes, such as *Jarid2*, *Cdx2 *and *Sox2 *have been identified previously as OCT4 or NANOG targets [12]. Additionally, as seen in both the human and mouse ES cell studies, OCT4 and NANOG bind to their own as well as each other's promoters [10,12,15,16]. We also observed a substantial overlap between the OCT4 and NANOG-bound genes, where 373 gene promoters were occupied by both these factors (Figure 1g). Together, these binding data support prior models suggesting that OCT4 and NANOG act together to maintain ES cell pluripotency by promoting self-renewal and by regulating a number of developmentally important genes.

**Figure 2:**
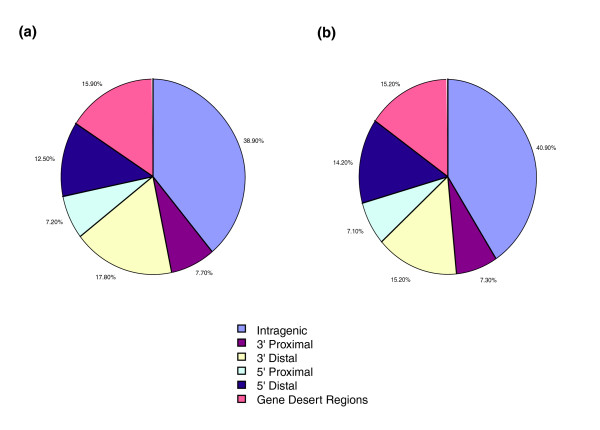
Genomic distribution of **(a) **OCT4 and **(b) **NANOG binding sites on mouse chromosome 19, obtained by ChIP-chip analyses.

Given that it has been reported that a significant number of binding sites may be located outside of promoter regions [12], we next hybridized the OCT4 and NANOG ChIP samples to chromosome arrays that tiled the entire non-repeat portion of mouse chromosome 19 [Arrayexpress: A-MEXP-956]. Binding events were analyzed similarly to the promoter arrays and occupied regions were identified using the same criteria (Additional data files 8, 15 and 17). In addition to promoter regions bound by OCT4 and NANOG, this analysis revealed OCT4 and NANOG binding sites that were undetectable on the promoter arrays (Figure 1e,f). Bound regions were classified in relation to the nearest gene within 100 kb as: 5' proximal (0-10 kb upstream), 5' distal (10-100 kb upstream), 3' proximal (0-10 kb downstream), 3' distal (10-100 kb downstream) and intragenic (within the gene). Sites that were located >100 kb away from the nearest gene were classified as gene desert regions. We identified 208 binding events for OCT4 and 381 for NANOG using the chromosome array. For both factors we observed similar trends in distribution of these binding sites across chromosome 19 (Figure 2). Among OCT4 targets, 38.9% of the sites were in intragenic, 7.7% in the 3' proximal, 17.8% in the 3' distal, 7.2% in 5' proximal, 12.5% in 5' distal, and 15.9% in gene desert regions. Following a similar distribution, the NANOG data showed 40.9% of the binding sites in intragenic, 7.3% in the 3' proximal, 15.2% in the 3' distal, 7.1% in 5' proximal, 14.2% in 5' distal, and 15.2% in gene desert regions. These results show that OCT4 and NANOG targets are located across different genomic regions, and such extensively tiled arrays can be used to obtain more detailed binding data on a genome-wide scale. Additionally, the finding that approximately 40% of the binding sites were found in intragenic regions is also in concordance with earlier observations made in the ChIP-PET study for both OCT4 and NANOG, indicating that the chromosome 19 array results are representative of the binding distribution in the genome.

### OCT4 and NANOG ChIP-PET data

In order to compare genomic targets across different platforms, we re-analyzed previously reported ChIP-PET experimental data for OCT4 and NANOG [12]. In the ChIP-PET method, immuno-enriched DNA fragments are cloned into a plasmid library, which is then transformed into one containing concatenated signature paired-end ditag sequences. The DNA fragments or binding sites are subsequently sequenced and the reads are mapped to the mouse genome. All binding sites were first classified relative to the nearest gene (as intragenic, 5'distal, 5' proximal, 3' distal, 3' proximal and gene desert regions), according to the criteria described earlier. Next, we performed GO analyses on the ChIP-PET targets in each of these regions. We observed that similar to ChIP-chip data, both OCT4 and NANOG binding targets had a significant representation of genes encoding transcription factors and regulators of cell fate (Additional data file 7).

In order to analyze the ChIP-PET and ChIP-chip data together, all raw ChIP-PET sequence reads were re-mapped to the same version of the mouse genome (mm6) used in the ChIP-chip experiments. The sequence reads were between 34 and 36 bp, and only those that had at least 34 matched base pairs and a gap-length of 10 bp were considered to be uniquely mapped to the mouse mm6 genome. Out of 951,437 OCT4 reads, 198,802 (20.9%) could be uniquely mapped. Similarly, among 624,237 NANOG reads, 333,248 reads (53.4%) could be mapped uniquely to the genome. Importantly, the methods and criteria used to remap data to a different genome version will provide a useful resource for performing such analyses with other sequencing based platforms that use other genome versions.

In ChIP-PET experiments, a minimum number of overlapping sequence reads was used as a criterion for identifying binding events. A region was considered occupied by OCT4 and NANOG if it had at least four or three overlapping sequence reads, respectively. In order to analyze our ChIP-chip findings in relation to these data, we examined only those ChIP-PET reads that had corresponding regions represented on the mouse promoter arrays (576 for OCT4 and 924 for NANOG). Additionally, for chromosome 19, 90 OCT4 targets and 224 NANOG targets could be remapped for the ChIP-PET data.

### Examination of ChIP-chip and ChIP-PET bound regions

**Figure 3:**
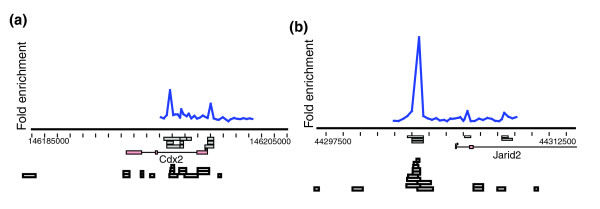
GSE Spatial Visualizer snapshots showing illustrative examples of ChIP-chip and ChIP-PET data for **(a) **OCT4 and **(b) **NANOG. The fold enrichment for a single ChIP-chip replicate (for OCT4 or NANOG) is shown against the genomic coordinate scale for the gene (in base pairs). The grey boxes represent the locations of 'bound regions' from each of the factor's three ChIP-chip replicates. The white boxes show the overlapping ChIP-PET reads for the displayed region. A 'bound region' in ChIP-PET experiments had four or more overlapping reads in OCT4, and three or more overlapping reads in the case of NANOG. Gene exons and introns are represented by pink boxes and solid horizontal lines, respectively. For each visualized gene, the transcriptional start site, direction of transcription and RefSeq annotation derived from the UCSC database are also specified.

To examine the binding events obtained by ChIP-chip and ChIP-PET, we used the 'Genomic Spatial Events' (GSE) Visualizer program [17] (Figure 3) (Additional data file 15). GSE is a Java software package, written to allow interactive browsing of ChIP-chip and ChIP-PET data, and genome annotations, from a remote database over a network connection (software for this program is available upon request). It handles datasets that are simultaneously mapped against multiple genome builds, a requirement for any system that is to compare new experimental data against older datasets. The software is built to run on multiple platforms, and also provides a software interface for custom-written analysis modules. The locations of bound probes from replicate ChIP-chip experiments, as well as the overlapping ChIP-PET reads for the respective regions, could be simultaneously visualized using the program. Therefore, this tool provides an important resource to compare data from multiple sources at a variety of genomic scales. It can also be utilized in the future for such purposes as data using other technologies become available for expanding the ES cell transcriptional circuitry.

**Figure 4:**
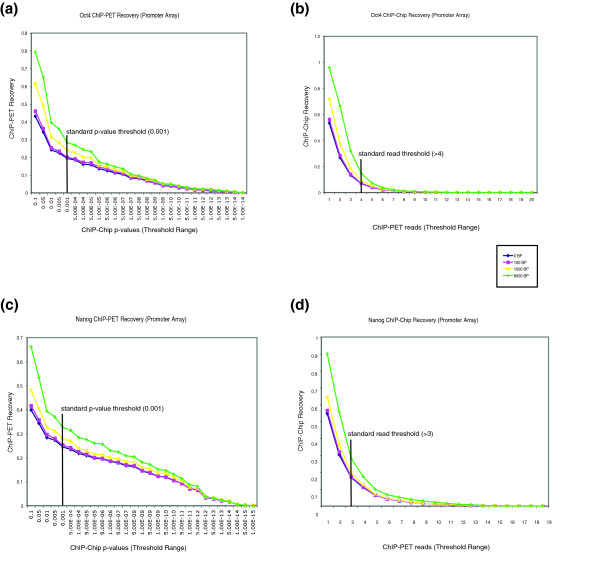
OCT4 and NANOG promoter array recovery curves. **(a, c) **The OCT4 (a) and NANOG (c) ChIP-PET recovery curves for the promoter arrays. These represent the fraction of ChIP-PET recovery under a range of ChIP-chip *p*-value cut-offs. **(b, d) **OCT4 (b) and NANOG (d) ChIP-chip recovery curves. These show the ChIP-chip percent recovery at varying ChIP-PET read thresholds. In all cases, recovery curves are made for a variety of distances (0-8 kb) permitted between a ChIP-chip peak and ChIP-PET read for them to be considered 'overlapping'.

**Figure 5:**
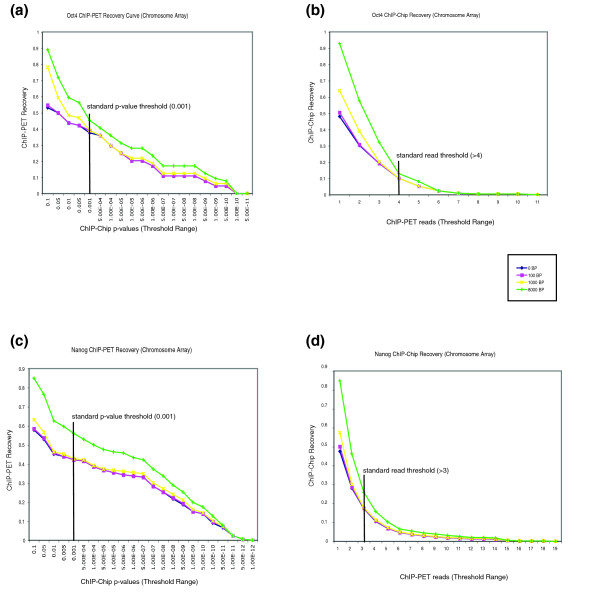
OCT4 and NANOG chromosome array recovery curves. **(a, c) **The OCT4 (a) and NANOG (c) ChIP-PET recovery curves for the chromosome array. These represent the fraction of ChIP-PET recovery under a range of ChIP-chip *p*-value cut-offs. **(b, d) **OCT4 (b) and NANOG (d) ChIP-chip recovery curves. These show the ChIP-chip percent recovery at varying ChIP-PET read thresholds. In all cases, recovery curves are made for a variety of distances (0-8 kb) permitted between a ChIP-chip peak and ChIP-PET read for them to be considered 'overlapping'.

In order to determine how the analysis methods and threshold criteria in ChIP-chip and Chip-PET experiments influenced the overall concordance between datasets, we examined the data by generating 'recovery curves' (see Materials and methods; Figures 4 and 5). A binding event in one experiment was considered 'recovered' by (or overlapping with) a similar event in a second experimental type if both events were within a fixed genomic distance (recovery distance) from each other. A typical *p*-value threshold of 0.001 was used initially to determine significant binding events in ChIP-chip experiments, and a minimum number of 'overlapping sequence reads' was used to establish bound regions in ChIP-PET experiments (four or more overlapping reads for OCT4 targets, and three or more overlapping reads for NANOG). We generated two types of recovery curves to analyze the ChIP-chip and ChIP-PET data. The ChIP-PET recovery curve examined the fraction of ChIP-PET regions overlapping with the ChIP-chip data at a wide range of *p*-value thresholds for the ChIP-chip experiments. In this instance, the threshold criteria were kept constant for the ChIP-PET experiments, and the ChIP-PET recovery (y-axis) was plotted against a range of ChIP-chip *p*-values (x-axis). Conversely, the other type of curve represented the ChIP-chip recovery at varying ChIP-PET 'overlapping read' threshold values. The ChIP-chip *p*-value threshold was kept constant at 0.001, and the ChIP-chip recovery (y-axis) was examined at different numbers of ChIP-PET sequence reads (x-axis). We examined each type of curve under a range of recovery distances, as binding events identified by both methods may not have exact overlaps due to differences in sample processing and technologies.

Not surprisingly, we observed that the recoveries of OCT4 and NANOG targets obtained by one experimental method increased as the threshold value for the other method was relaxed. The recoveries also increased as the distance permitted between a ChIP-chip peak and corresponding ChIP-PET peak was increased (Figures 4). As an example of these results, converting the recoveries into percentages, among OCT4-bound regions, 24% of the peaks identified by ChIP-PET (>4 reads) were recovered in the ChIP-chip data (*p*-value < 0.001) within a distance of 1 kb. Conversely, using the same thresholds, 9.3% of the OCT4-bound peaks found by ChIP-chip were recovered in the ChIP-PET data (Additional data file 9). From the NANOG data we observed that 28.1% of the ChIP-PET peaks (>3 reads) were recovered in ChIP-chip bound regions (*p*-value < 0.001) within a 1 kb area. Conversely, the ChIP-chip percent recovery from ChIP-PET bound regions (>3 reads) was 19.5% (Additional data file 10). Therefore, these recovery curves illustrate the importance of recovery distance and threshold calibration in examining data from different sources.

Similar analyses were performed using the mouse chromosome 19 data and corresponding ChIP-PET regions. We noted that the amount of overlap between ChIP-PET and ChIP-chip increased with the more extensively tiled arrays (Figure 5). This is because many targets identified in ChIP-PET experiments would not be identified by the promoter arrays, since regions outside of the promoter were not represented on these arrays. In summary, the OCT4 and NANOG ChIP-chip and ChIP-PET datasets show that the recovery among datasets varies as any of the threshold criteria for binding events are altered. Further evaluation of the binding events identified through both techniques, by genetic manipulation of the corresponding genes in ES cells, will lend better insight into the genes responsible for maintaining pluripotency.

Previous reports have suggested that a lack of concordance between array- and sequencing based technologies may also be due to the repeat-masking feature of tiled microarrays as well as a sequencing depth issue with ChIP-PET [11,14]. Since 99% of the ChIP-chip probes on our promoter arrays do not have any major overlaps with repeat regions, and only 8.1% of all ChIP-PET sequences fall in repeat-masked regions, we do not expect the results of this study to change by any significant degree if this small fraction of ChIP-PET sequences is removed from the analysis. In order to further examine the sequencing depth issue, the ChIP-chip and ChIP-PET data on chromosome 19 were used to perform a sequence-depth analysis to examine the changes in ChIP-chip recovery as increasing numbers of ChIP-PET sequences are randomly sampled (Additional data files 3 and 14). According to our observations for both OCT4 and NANOG, the number of ChIP-chip targets recovered increased with the number of ChIP-PET reads sampled, and did not approach a saturation point, even when all ChIP-PET reads for chromosome 19 were sampled. This result suggests that the lack of recovery of ChIP-chip targets in the ChIP-PET data can, at least in part, be explained by a lack of depth in sequencing.

### Differentially regulated targets of OCT4 and NANOG

Since protein-DNA binding alone is not indicative of a regulatory event, the expression of OCT4 and NANOG binding targets obtained through ChIP-chip and ChIP-PET was compared by comparing binding data with previously published OCT4 and NANOG RNA interference (RNAi) gene expression profiles in ES cells [12]. The expression levels of targets determined exclusively by either technique, and those overlapping in both, were examined in *Oct4 *or *Nanog *knockdown ES cells (Table 1; Additional data files 11, 12 and 15) We found that among the OCT4-bound targets (with corresponding Affymetrix probes) determined only by ChIP-chip, 33.9% were differentially expressed upon *Oct4 *knockdown. Similarly, 29% of the OCT4 targets detected solely by ChIP-PET were differentially regulated. Interestingly, for OCT4 targets obtained by both ChIP-PET and ChIP-chip, 70.3% showed changes in gene expression upon downregulation of OCT4. In *Nanog *knockdown ES cells, 21.4% of the targets determined solely by ChIP-chip, and 14.8% identified only by ChIP-PET were differentially expressed compared to normal ES cells. This percentage increased for targets that were identified by both techniques, where 33.5% were differentially regulated upon *Nanog *knockdown. These analyses also showed that among the differentially regulated targets of NANOG, the distribution between up- and downregulated genes upon *Nanog *knockdown was approximately equal. However, in the case of OCT4 regulated targets, a larger percentage of genes was downregulated (60.4%) upon *Oct4 *knockdown. These results suggest that both NANOG and OCT4 can potentially activate or repress their binding targets. Therefore, these analyses have revealed a higher-value set of OCT4- and NANOG-regulated genes, by collectively examining the targets identified by ChIP-chip and ChIP-PET.

**Table 1:** Differential expression of OCT4 and NANOG targets in RNAi experiments

Method for determining bound targets	Percent of differentially expressed OCT4 targets on OCT4 knockdown	Percent of differentially expressed NANOG targets on NANOG knockdown
ChIP-chip only	33.9% (390/1,151)	21.4% (192/898)
ChIP-PET only	29% (114/393)	14.8% (91/616)
ChIP-chip + ChIP-PET	70.3% (83/118)	33.5% (73/218)
Percent of regulated genes		
Upregulated	39.4%	50.6%
Downregulated	60.6%	49.4%

The functional relevance of the ChIP-chip and ChIP-PET data, as examined by GO analyses, had revealed that the OCT4 and NANOG bound regions were significantly enriched for transcriptional and developmentally important regulators of gene expression. Similar observations had been made earlier for these factors in human ES cells as well [10]. For instance, among genes that displayed changes in expression levels upon *Oct4 *RNAi-mediated knockdown, certain genes, including *Sox2 *and *Rif1*, which have important roles in development, were bound by OCT4 in both ChIP-chip and ChIP-PET experiments. However, other genes that play a part in cell-fate determination, such as *Gdf3 *and *Notch4 *[18,19], were bound by OCT4 only in the ChIP-chip experiments. A separate set of differentially expressed OCT4 targets, including *Yap1 *and *Foxd3*, which have been shown to have developmentally important roles [20,21], were obtained only in the ChIP-PET data. Similarly, observations were made in the *Nanog *RNAi knockdown data, which showed changes in expression of target genes identified both exclusively and collectively by the two technologies. Therefore, combining the binding data obtained by both techniques, along with gene expression data, has provided a more detailed overview of the factors involved in the ES cell transcriptional circuitry. Further genetic studies of these regions will lend deeper insight into the mechanisms governing ES cell biology.

## Discussion

ChIP-based technologies are being used extensively in identifying protein-DNA interaction networks in a variety of cell types and a number of varying conditions. In particular, ChIP-PET and ChIP-chip have been used to identify the mouse and human ES cell transcriptional circuitries, which are largely regulated by the key pluripotency factors OCT4 and NANOG. Although each ChIP-based technology used in the identification of these networks has its distinct advantages, we find substantial differences in the data derived through these different experimental methods. Recent technological comparisons have shown differences in the results obtained by these methods, and illustrated the need to use these data in a complementary manner [14]. We have used ChIP-chip to uncover genomic regions bound by OCT4 and NANOG in mouse ES cells, and expanded on previously published ChIP-PET results, and find a large number of binding sites identified exclusively by each technique. Therefore, using these data in a complementary fashion provides a more detailed overview of the OCT4 and NANOG transcriptional networks.

We analyzed our ChIP-chip results for OCT4 and NANOG in relation to existing ChIP-PET data. Since the criteria for identification of genomic targets is different between platforms, the datasets obtained by the two methods were examined against each other under an exhaustive range of significance values. Recovery curves were used to measure the recovery of targets obtained by keeping the binding threshold for one technique constant and varying the threshold values for the other method. As expected, for both OCT4 and NANOG targets, the ChIP-PET recovery decreased as the ChIP-chip *p*-value threshold was made more stringent. A similar trend was observed for the ChIP-chip recovery when the ChIP-PET read stringency was increased. Additionally, at the same thresholds, this overlap decreased when the recovery distance permitted between a ChIP-chip peak and ChIP-PET peak was narrowed. Therefore, these recovery curves revealed the necessity of recovery distance calibration in examining binding experiments from multiple sources. Interestingly, we also observed that the amount of recovery between ChIP-chip and ChIP-PET data increased when the whole chromosome arrays were used. Therefore, the criteria used to determine a binding event, as well as the extent of genome coverage, had an effect on the overlap between the data obtained by the two methods. The recovery curves illuminated the sensitivity of recovery to distance threshold, and provided a useful means to examine the datasets relative to each other.

We combined the protein-DNA binding data with known *Oct4 *and *Nanog *RNAi expression profiling data in order to analyze the targets that are differentially regulated upon *Oct4 *or *Nanog *knockdown in ES cells. OCT4- and NANOG-bound regions uncovered by both technologies, as well as the ones obtained exclusively by each method, contained a number of differentially regulated genes. Many of these genes encode transcription factors and regulators of gene expression, which are important in development. For instance, the expanded OCT4 and NANOG regulatory network contained genes such as *Hoxa1*, *Foxd3*, *Msx2 *and *Hexb*, which showed changes in expression upon *Oct4 *or *Nanog *knockdown. These genes have been shown to be important in cell fate specification, and are involved in developmentally important signaling pathways. Such additional targets identified by each technique can be used to expand the ES cell transcriptional regulatory framework, and thereby provide more detailed groundwork to understand pluripotency mechanisms. Further genetic manipulations of each of these genes in ES cells would be necessary to independently validate their contributions to pluripotency.

Although both ChIP-chip and ChIP-PET technologies have been useful in studying protein-DNA interactions on a genome-wide scale, each method has its set of limitations. In ChIP-chip, our observations are restricted to regions tiled on the array platform, and the resolution is limited by the size of the probes, their spatial distribution, as well as the average fragment length of sonicated DNA hybridized to the arrays. In ChIP-PET experiments, the bacterial cloning and sequencing steps, as well as mapping issues, introduce scope for error. We feel that a combination of more stringent mapping criteria and the inherent noise in the sequencing procedure may be responsible for the number of sequence reads that did not match perfectly to the genome. Moreover, as indicated by our sequence-depth analysis, the number of sequences obtained from ChIP-PET experiments can be a limiting factor, since more binding targets can be recovered through greater depth in sequencing. Additionally, as in the case of ChIP-chip experiments, the resolution of binding is limited by the average DNA fragment size used in the ChIP experiment. We observed some of these limitations in this study since there was a significant number of OCT4 and NANOG targets that had been identified by ChIP-PET, and did not have corresponding probes tiled on the arrays used in the ChIP-chip experiments. Apart from these limitations, it is also important to consider that binding sites may be differentially occupied at different times in the cell cycle since the chromatin state changes at different times [22]. However, since it is currently not feasible to culture ES cells in a synchronized manner, such genome-wide analyses should be done with this caveat in mind. In addition to this, another limitation to these studies is that the processing of ES cell samples can vary between different laboratories and also between different batches of serum used to culture these cells. Finally, different binding results may be obtained due to differences in ES cell strains. Therefore, with the availability of binding information from different cell strains [11], we can begin to address such issues.

Apart from ChIP-chip and ChIP-PET, other ChIP based methodologies, such as ChIP-SACO (serial analysis of chromatin occupancy) [23] and STAGE (sequence tag analysis of genome enrichment) [24], have been used to determine protein-DNA interactions on a genome-wide scale. Most recently, ChIP-Seq [25], a sequencing based technology, has aimed to address many of the issues, such as genome coverage, sequencing depth and binding resolution, which are encountered by other currently used techniques. With this rapid change in technologies, it will be important to investigate the results obtained from these techniques and incorporate them into our current understanding of regulatory networks. Importantly, the use of multiple techniques has been shown to produce variations in the information obtained through individual platforms [14]. Using the data obtained through these different methodologies in a complementary fashion provides a more thorough foundation for further investigating these networks.

## Conclusion

The results of this study provide a useful way to integrate protein-DNA interaction data that are obtained by different techniques. We have used this to expand our current knowledge of the mouse ES cell regulatory network that is orchestrated by the transcription factors OCT4 and NANOG. Although both the ChIP-chip and ChIP-PET technology platforms identified different sets of binding events, a considerable number of these events represented genes that were regulated by OCT4 and NANOG. Since a number of these genes have known roles in important developmental pathways and in cell-fate specification, it will be interesting to explore their biological roles with respect to ES cell pluripotency. Therefore, this expanded network provides a stronger foundation to further examine biochemical and genetic interactions that regulate stem cell properties. Moreover, the methods described to compare datasets from different platforms would be very useful as data from newer technologies, such as Chip-Seq, become available. Since ES cells are a model system for studying developmental processes, and are thought to hold great promise in regenerative medicine, it will be important to gain a thorough understanding of the means by which a stem cell maintains its identity, and how it can be directed to form different cell types. Our work will allow for a more detailed examination of the components of this expanded stem cell circuitry and will lend better insight into the mechanisms of pluripotency.

## Materials and methods

### ES cell culture

V6.5 murine ES cells (genotype 129SvJae × C57BL/6; male) were grown at 5% CO_2 _at 37°C on gelatinized tissue-culture plates. They were grown in DMEM (Gibco, 11965-118, Grand Island, NY, USA) with 15% fetal bovine serum (FBS; Hyclone, Lot No. ARC26080, South Logan, UT, USA), Leukemia inhibitory factor, 1% penicillin/streptomycin (100× stock from Gibco, 15140-122), 1% L-glutamine (200 mM; Gibco, 25030-081), 1% non-essential amino acids (100× stock from Gibco, 11140-050) [26]. Since the replication time of ES cells can vary with different batches of serum, the doubling time of ES cells grown with this batch of serum was calculated to be approximately 16 hours. This doubling time was comparable to that obtained with other lots of FBS (Hyclone, Lot numbers ASJ30355 and ASB28896). Moreover, as an additional control, KH2 ES cells [27] were also cultured in these different batches of FBS, and showed similar doubling times as v6.5 ES cells The cells were grown without irradiated mouse embryonic fibroblasts prior to the ChIP analyses in order to minimize contamination from feeders.

### Antibodies

For ChIP experiments, we used anti-NANOG (Bethyl, BL-1162, Montgomery, TX, USA) and anti-OCT4 (SantaCruz, sc-8628X, Santa Cruz, CA, USA), which have been previously characterized for immunoprecipitation.

### Chromatin immunoprecipitation

The ChIP protocol was similar to previously published studies [10,28-30]. Briefly, for each location analysis reaction, approximately 1.5 × 10^8 ^ES cells were grown at 70-80% confluency. The cells were cross-linked by adding fresh 11% formaldehyde solution to the ES media for 10 minutes at room temperature. The cells were washed twice with 1× phosphate-buffered saline and scraped off the plates, pelleted and stored at -80°C. They were then lysed and sonicated to solubilize chromatin and shear the cross-linked DNA. Sonications were performed with a Misonix Sonicator 3000 and sonicated at power 7 for 12 × 30 second pulses (60 second pause between pulses) at 4°C while the samples were immersed in a water bath. The whole cell extract was incubated overnight on a rotating platform at 4°C with 100 μl of Dyna1 Protein G magnetic beads, blocked with 0.05% bovine serum albumin/phosphate-buffered saline and pre-incubated for 6 hours to overnight with 10 μg of antibody of choice. The beads were washed five times with radio immunoprecipitation assay (RIPA) buffer and once with TE containing 50 mM NaCl. Bound protein-DNA complexes were eluted off the beads in elution buffer by occasional vortexing and heating at 65°C overnight. Whole cell extract (saved from the sonication step) was treated similarly for cross-link reversal. Following treatment with RNaseA and Proteinase K, the immunoprecipitated and whole cell extract DNA was purified by phenol:chloroform:isoamyl alcohol extraction. The DNA was blunted-end ligated to a universal linker and amplified using a two-stage expansion PCR protocol (three reactions per sample were done for the second expansion and combined). The amplified immunoprecipitated DNA and whole cell extract DNA were labeled with Cy5 and Cy3 fluorophores, respectively, using Invitrogen random primer labeling kits (Invitrogen, Carlsbad, CA, USA). DNA (1 μg) was used in a labeling reaction and three labeling reactions were done per sample and combined after purification. The labeled DNA was purified using Invitrogen BioPrime Array CGH module purification kit. Five micrograms each of immunoprecipitated and whole cell extract DNA was combined along with mouse Cot-1 DNA and hybridized to each of the arrays in Agilent hybridization chambers for 40 hours at 65°C using the Agilent hybridization protocol and reagents for 244 K arrays (Agilent, Santa Clara, CA, USA). Arrays were then washed and scanned as previously described. Three biological replicates were done for each transcription factor in order to determine statistical significance for binding targets.

### Analysis of ChIP-chip data

Three biological replicates were examined for both OCT4 and NANOG using mouse promoter arrays. The same samples were also hybridized to mouse chromosome arrays. A probe was marked 'bound' in a particular replicate if its Rosetta *p*-value was below a pre-determined cutoff (usually 0.001, although this was systematically varied for the recovery curves). For each bound probe, a region around the probe was marked 'bound' extending to the nearest unbound probe (or 1 kb, if the nearest probe was more than 1 kb away). A region was marked as a 'peak' if it was bound in all three replicates.

The list of micro-RNAs to be analyzed was taken from version 8.0 of miRBase [33], which were mapped against NCBI build 34 of the mouse genome (mm6). We retained for analysis only those micro-RNAs which had at least three probes from the promoter array design mapped to within 4 kb of the miRBase annotation. Of the original 267 miRBase annotations, 258 satisfied this requirement.

### Mapping of ChIP-PET sequences to the mouse genome

Two sets of previously published ChIP-PET experiments for OCT4 and NANOG were examined. These reads were mapped to the Mouse mm6 genome sequence in order to be consistent with the genome version used for the arrays in the ChIP-chip experiments. The mapping was done using the BLAT sequence alignment tool, with a step size of 5 and tile size of 11.

We received two FASTA files of sequences, each between 34 and 36 bp in length. Only those sequence hits reported by BLAT that had at least 34 matched bases (corresponding to 0, 1, or 2 mismatches), and a gap-length of at least 10 bp, were retained. Any sequences with more than one retained hit to the mm6 genome were filtered out. The locations of the unique hits for the sequences that remained were stored in a database, and used for comparison with the ChIP-chip peaks.

### Comparison of ChIP-chip to Chip-PET data

In order to examine if a bound region from ChIP-chip was 'matched' to another bound region from ChIP-PET, and vice versa, a simple genomic distance threshold was determined. If the two regions were on the same chromosome, and if the edge of one region was within the recovery distance from the edge of the other, then they were considered 'matched'. The typical recovery distance used was 1 kb, although this was varied systematically for the recovery curves. A recovery distance of '0' represents only strictly overlapping regions that were matched to each other.

### Recovery curves

Recovery curves were produced for promoter and chromosome array data for OCT4 and NANOG. One curve was for the ChIP-chip (*p*-value) cutoff, and the other for the ChIP-PET (overlapping region) cutoff. In the ChIP-PET recovery curves, fixed cutoffs of four overlapping reads for *Oct4 *and three overlapping reads for *Nanog *were used to determine a 'background set' of ChIP-PET bound regions, as described above. At that ChIP-PET cut-off, the number of ChIP-chip bound regions was calculated for a range of *p*-value cut-offs. The percentage of background ChIP-PET bound regions that were matched by at least one ChIP-chip bound region, at each *p*-value threshold, was graphed. This was repeated for several different matching distances.

For calculating the ChIP-chip recovery curve, this process was repeated by holding the ChIP-chip binding threshold constant. A background set of bound regions using ChIP-chip was calculated using a *p*-value threshold of 0.001. The threshold of overlapping reads used to call bound regions in ChIP-PET was varied from 1 to an upper limit where no matched regions were called. At each threshold, the fraction of the background ChIP-chip bound regions matched by one of the ChIP-Pet regions was calculated. This process was also repeated for several different matching distances.

### Sequence depth analysis

In order to carry out the sequence-depth analysis using data from chromosome 19, the total number of ChIP-PET reads for OCT4 and NANOG were calculated. For OCT4, 8,675 reads, and for NANOG, 5,233 reads were mapped to chromosome 19. The number of ChIP-chip bound regions recovered for both proteins was determined for differently sized subsets of these ChIP-PET reads. We started with a random subset that sampled 10% of the reads, and determined the ChIP-chip recovery for that sample. This analysis was done for increasingly large subsets of ChIP-PET reads in increments of 10%, until all ChIP-PET reads on chromosome 19 were sampled. For each subset size, the same number of reads was randomly sampled ten times to calculate the average ChIP-chip recovery and standard deviation in each case.

### Expression analysis of OCT4 and NANOG targets

We wanted to determine the relevance to pluripotency for OCT4 and NANOG binding targets obtained by ChIP-chip or ChIP-PET. Previously published *Oct *and *Nanog *RNAi gene expression profiles in ES cells were used for this analysis [12]. These experiments produced sets of Affymetrix probes, which were differentially regulated in one or more of the replicates. Those probes were matched to sets of gene names using an Affymetrix-provided probe annotation file. A gene was determined as differentially regulated in either the *Oct4 *or the *Nanog *knockdown experiments if it was associated with any differentially regulated probe in the replicates of that factor's experiments. A gene annotation was called 'bound by ChIP-chip' if there was a ChIP-chip peak within ± 4 kb of the gene annotation's start site. Equivalently, a gene annotation was also bound by ChIP-PET if a ChIP-PET peak fell within the same distance of the annotated start site (Additional data file 4). This allowed us to divide the set of differentially regulated gene annotations into four categories: bound in both experimental types, bound in neither, and bound in one (but not the other).

### Gene ontology analysis

A set of bound RefSeq gene identifiers was compiled for the OCT4 and NANOG bound genes according to a standard cutoff (*p*-value < 0.001 for ChIP-chip, and a maximum distance of 4 kb from the gene start site for any annotated gene). The sets of RefSeq identifiers were converted into sets of 'known gene' gene symbols, using the kgXref table of the UCSC Genome database [34]. A set of GO categories was downloaded from the Gene Ontology Project [31], and an updated set of mouse annotations was downloaded from European Bioinformatics Institute's (EBI) Gene Ontology Annotation Project [32]. Since the GO categories are structured as a directed graph, we propagated the annotations backwards through the graph; a gene symbol was marked as annotated to a GO category if the annotation was contained in the EBI dataset, or if the GO category was the ancestor of a GO category to which the gene symbol had already been assigned. A background set of gene symbols was assembled from the corresponding 'known gene' symbols for any RefSeq gene identifier that was tiled on the ChIP-chip array design. Given foreground sets of genes defined by the ChIP binding experiments, a background set of arrayed gene symbols, and a set of EBI-derived GO category annotations for each symbol, we calculated a list of GO categories ranked by their enrichment in each of the four foreground gene sets. Enrichment was calculated using the *p*-value of the hypergeometric probability for the overlap of each test set with each GO category.

## Abbreviations

ChIP, chromatin immunoprecipitation; EBI, European Bioinformatics Institute; ES, embryonic stem; FBS, fetal bovine serum; GO, Gene Ontology; GSE, Genomic Spatial Events; PET, paired-end ditag; RNAi, RNA interference.

## Authors' contributions

DM conducted all experiments for the paper and wrote the manuscript. TWD carried out data analyses and assisted with writing the manuscript. LAB conceived of the project and provided advice on the manuscript. RAY, DKG and RJ provided advice and funding for this work.

## Additional data files

The following additional data are available with the online version of this paper. Additional data file 1 is a figure depicting OCT4 ChIP-chip enrichment plots. Additional data file 2 is a figure depicting NANOG ChIP-chip enrichment plots. Additional data file 3 is a figure showing sequence depth analysis for OCT4 and NANOG targets on chromosome 19. Additional data file 4 is a figure depicting an example of inconsistency in combining expression-profiling data with binding information from ChIP-chip and ChIP-PET. Additional data file 5 is a table of protein coding gene promoter regions that were bound by OCT4 and NANOG in ChIP-chip and ChIP-PET experiments. Additional data file 6 is a table of micro-RNA gene promoter regions that were bound by OCT4 and NANOG in ChIP-chip experiments. Additional data file 7 contains tables of GO analyses for OCT4 and NANOG bound regions in ChIP-chip and ChIP-PET experiments. Additional data file 8 contains tables of chromosome 19 regions that are bound by OCT4 and NANOG in ChIP-chip experiments. Additional data file 9 contains tables listing OCT4 ChIP-chip and ChIP-PET recovery curve values for promoter and chromosome array experiments. Additional data file 10 contains tables listing NANOG ChIP-chip and ChIP-PET recovery curve values for promoter and chromosome array experiments. Additional data file 11 is a table of OCT4 bound genes that are differentially expressed upon *Oct4 *knockdown. Additional data file 12 is a table of NANOG bound genes that are differentially expressed upon *Nanog *knockdown. Additional data file 13 contains tables listing gene-specific PCR binding data for OCT4 and NANOG. Additional data file 14 contains tables showing sequence depth analysis for OCT4 and NANOG bound regions on chromosome 19. Additional data file 15 contains supplementary information on additional methods and materials and results. Additional data file 16 is a summary of promoter array ChIP-chip binding data and corresponding ChIP-PET binding data for OCT4 and NANOG. Additional data file 17 is a summary of chromosome array ChIP-chip binding data and corresponding ChIP-PET binding data for OCT4 and NANOG.

## Supplementary Material

Additional data file 1 Immunoprecipitate versus whole cell extract enrichment in three biological replicate samples is shown for (a) the two-slide set promoter arrays and (b) chromosome 19 arrays. Click here for file

Additional data file 2 Immunoprecipitate versus whole cell extract enrichment in three biological replicate samples is shown for (a) the two-slide set promoter arrays and (b) chromosome 19 arrays. Click here for file

Additional data file 3 Plots indicate the number of ChIP-chip targets recovered (y-axis) when different percentages of ChIP-PET sequences are randomly sampled. Error bars represent the standard deviation. Click here for file

Additional data file 4 The binding of NANOG at the REST gene promoter is identified by both ChIP-chip and ChIP-PET. However, the binding event detected in the ChIP-PET experiment is not within the region used for combining the expression profiling information (± 4 kb around the transcription start site). Consequently, the changed expression of REST after NANOG knockdown is associated with NANOG binding detected by ChIP-chip only and not ChIP-PET. Click here for file

Additional data file 5 Protein coding gene promoter regions that were bound by OCT4 and NANOG in ChIP-chip and ChIP-PET experiments. Click here for file

Additional data file 6 Micro-RNA gene promoter regions that were bound by OCT4 and NANOG in ChIP-chip experiments. Click here for file

Additional data file 7 GO analyses for OCT4 and NANOG bound regions in ChIP-chip and ChIP-PET experiments. Click here for file

Additional data file 8 Chromosome 19 regions that are bound by OCT4 and NANOG in ChIP-chip experiments. Click here for file

Additional data file 9 OCT4 ChIP-chip and ChIP-PET recovery curve values for promoter and chromosome array experiments. Click here for file

Additional data file 10 NANOG ChIP-chip and ChIP-PET recovery curve values for promoter and chromosome array experiments. Click here for file

Additional data file 11 OCT4 bound genes that are differentially expressed upon *Oct4 *knockdown. Click here for file

Additional data file 12 NANOG bound genes that are differentially expressed upon *Nanog *knockdown. Click here for file

Additional data file 13 Gene-specific PCR binding data for OCT4 and NANOG. Click here for file

Additional data file 14 Sequence depth analysis for OCT4 and NANOG bound regions on chromosome 19. Click here for file

Additional data file 15 Supplementary information on additional methods and materials and results. Click here for file

Additional data file 16 Summary of promoter array ChIP-chip binding data and corresponding ChIP-PET binding data for OCT4 and NANOG. Click here for file

Additional data file 17 Summary of chromosome array ChIP-chip binding data and corresponding ChIP-PET binding data for OCT4 and NANOG. Click here for file
