# Supplementary figures and images for: Analysis of the mouse embryonic stem cell regulatory networks obtained by ChIP-chip and ChIP-PET
Source: Genome Biol. 2008 Aug 13;9(8):R126. doi: 10.1186/gb-2008-9-8-r126 (PMC2575516; doi:10.1186/gb-2008-9-8-r126)

MATHUR\_FIG S1.

A

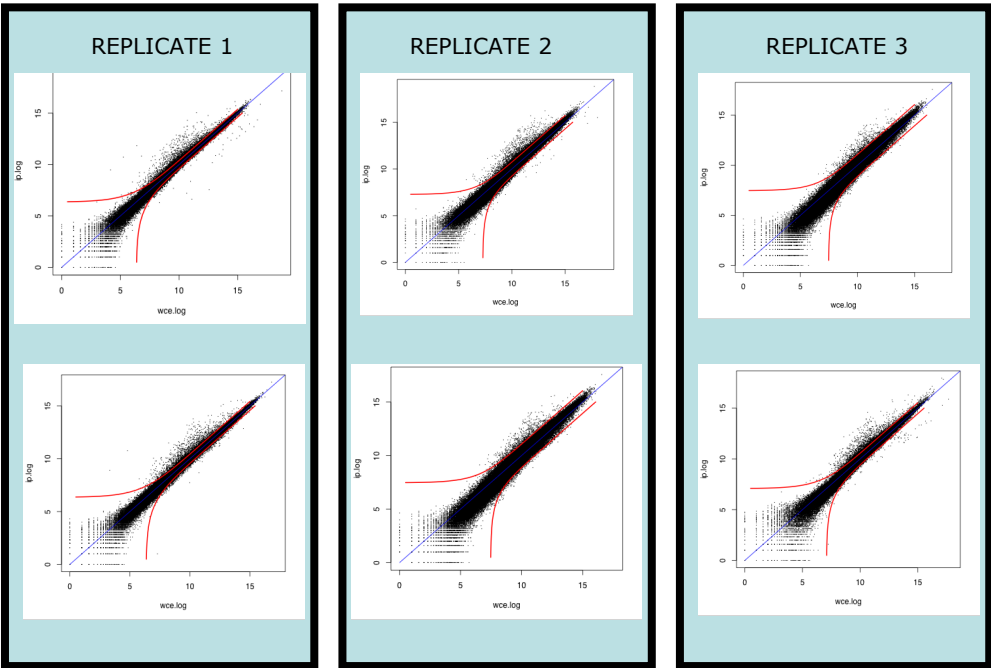

B

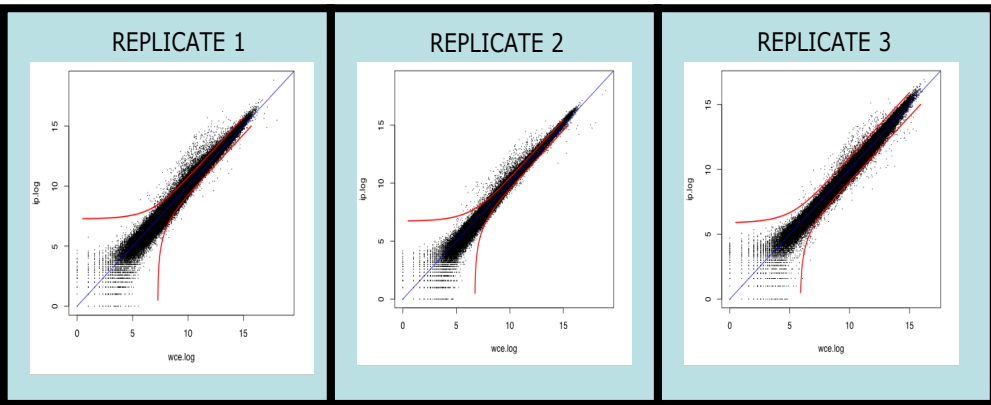

Supplement: Additional data file 1 — Immunoprecipitate versus whole cell extract enrichment in three biological replicate samples is shown for (a) the two-slide set promoter arrays and (b) chromosome 19 arrays. [file gb-2008-9-8-r126-S1.pdf]

MATHUR\_FIG S2.

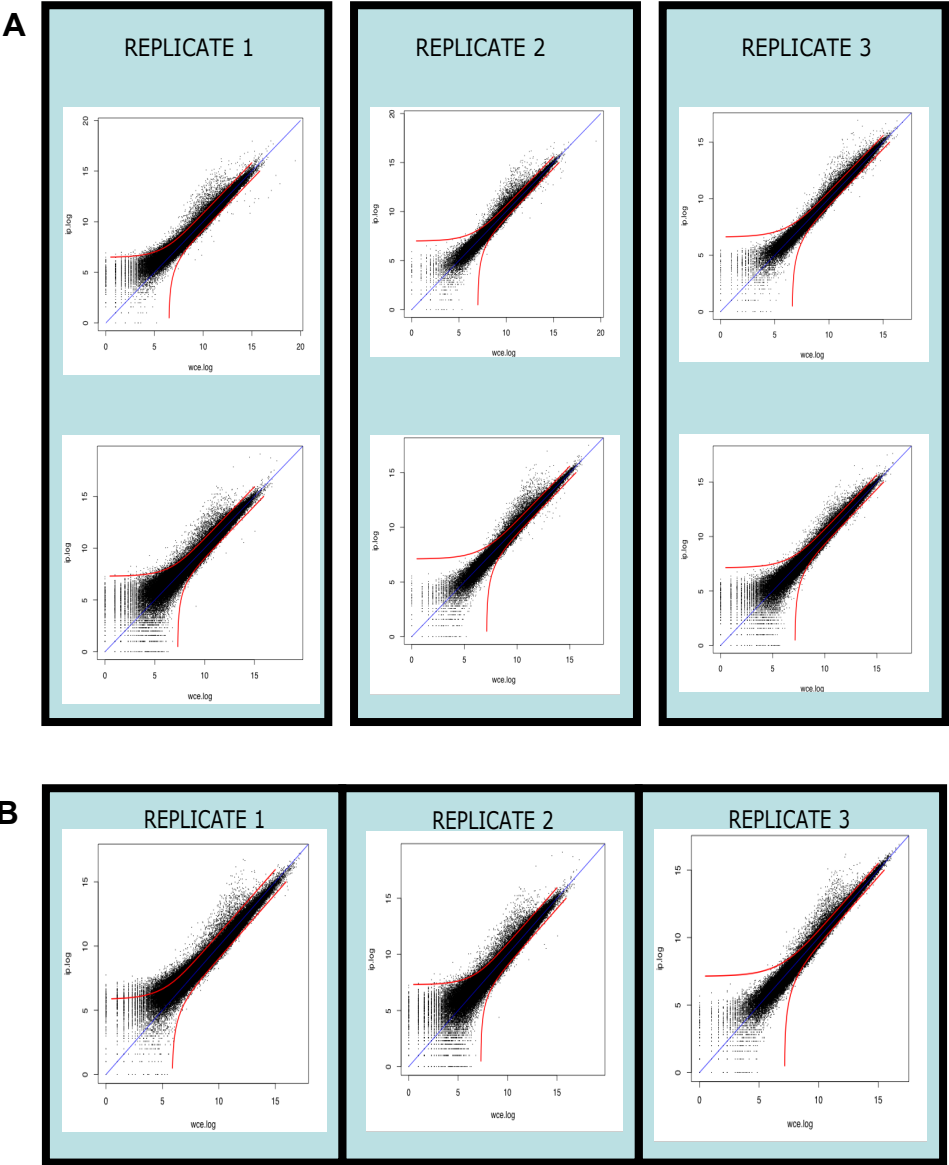

Supplement: Additional data file 2 — Immunoprecipitate versus whole cell extract enrichment in three biological replicate samples is shown for (a) the two-slide set promoter arrays and (b) chromosome 19 arrays. [file gb-2008-9-8-r126-S2.pdf]

Mathur\_Fig S3

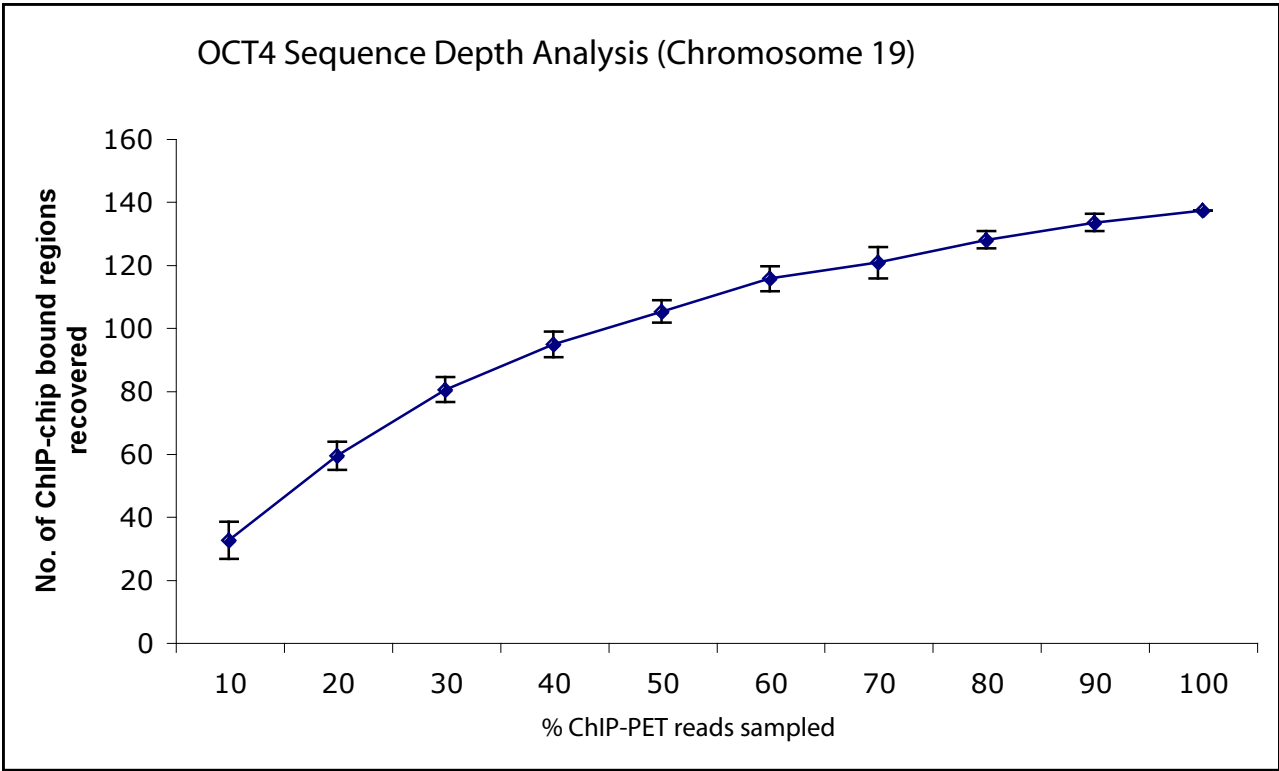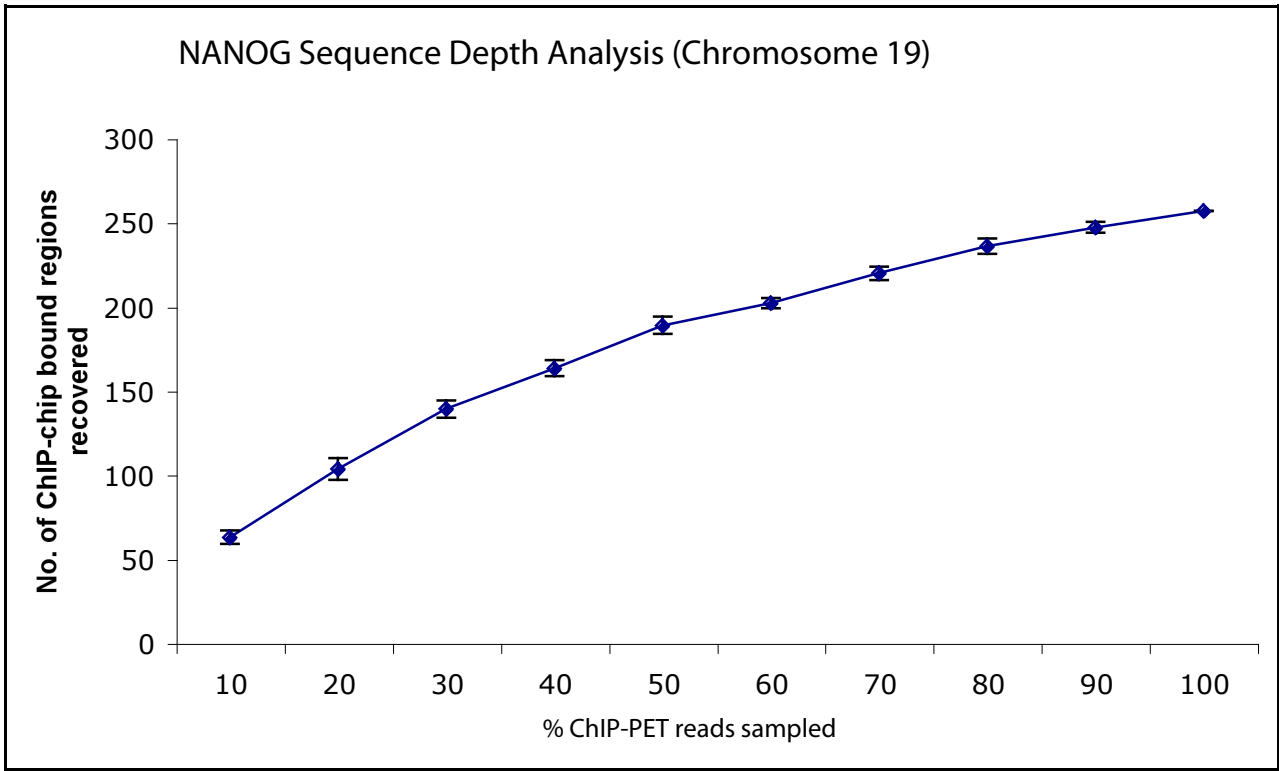

Supplement: Additional data file 3 — Plots indicate the number of ChIP-chip targets recovered (y-axis) when different percentages of ChIP-PET sequences are randomly sampled. Error bars represent the standard deviation. [file gb-2008-9-8-r126-S3.pdf]

MATHUR\_FIG S4.

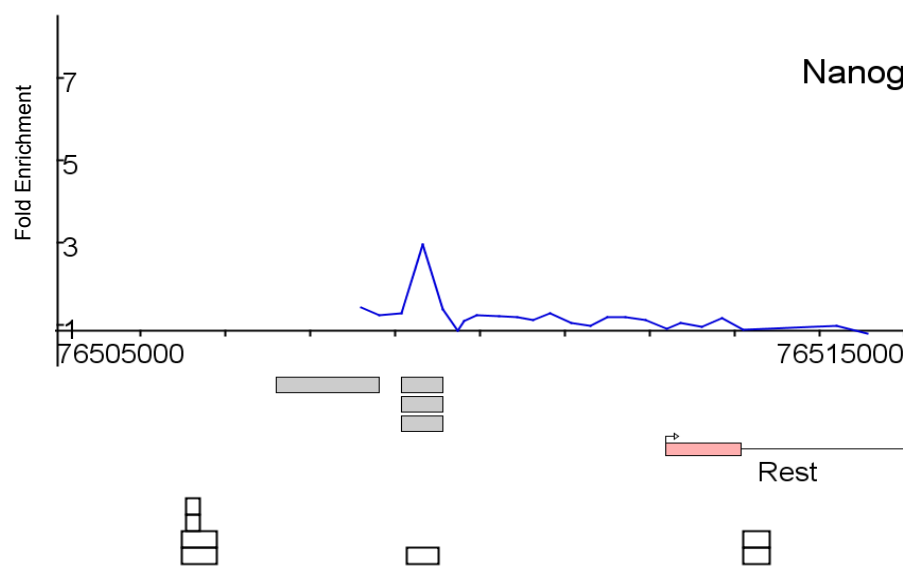

Supplement: Additional data file 4 — The binding of NANOG at the REST gene promoter is identified by both ChIP-chip and ChIP-PET. However, the binding event detected in the ChIP-PET experiment is not within the region used for combining the expression profiling information (± 4 kb around the transcription start site). Consequently, the changed expression of REST after NANOG knockdown is associated with NANOG binding detected by ChIP-chip only and not ChIP-PET. [file gb-2008-9-8-r126-S4.pdf]
