# Supplementary material for: Analysis of the mouse embryonic stem cell regulatory networks obtained by ChIP-chip and ChIP-PET
Source: Genome Biol. 2008 Aug 13;9(8):R126. doi: 10.1186/gb-2008-9-8-r126 (PMC2575516; doi:10.1186/gb-2008-9-8-r126)
Supplement: Additional data file 15 — Supplementary information on additional methods and materials and results. [file gb-2008-9-8-r126-S15.doc]

## Supplementary information

## *Supplementary note*

We have summarized the ChIP-chip promoter [see Additional data file 16] and chromosome array data [see Additional data file 17] and ChIP-PET raw data for OCT4 and NANOG. The ChIP-chip data for each region contains the location and value of each probe in that region (the first number is the locations, and the second number, marked with a ‘+’, is the offset of that probe within that region). Each probe is followed with its binding ratio in each of the three replicates. A bound probe is marked with a ‘*’. A second list represents a set of coordinates for each distinct ChIP-PET read that falls within that window. The numbers marked with a ‘+' represent the offset within that region.

## *Oligo-array design*

Two kinds of array platforms were employed in this study. One was a 2-slide mouse promoter array that is based on the 10-slide mouse promoter array set described in previous studies [1-3]. The arrays were manufactured at Agilent Technologies (www.agilent.com). The arrays include 19.993 features that include the promoters of all annotated genes in the NCBI Refseq database and miRNAs in miRBase. They also include promoters of alternate transcription start sites (TSSs). The oligos are 60-mers and span from 4kb upstream to 4kb downstream of the TSS at a density of one probe every 250 bp. Additionally the entire mouse *HoxA* cluster is also tiled on these arrays.

The second type of array was a whole chromosome array for the mouse chromosome 19. It also tiles the non-repeat portion of the ENCODE Design. Since ENCODE is a human project, analogous regions in mouse were mapped for this array. The oligo length and probe density is similar to the promoter arrays.

### Gene-specific PCR for OCT4 and NANOG bound regions

Gene Specific PCR was performed for three different sets of targets for both OCT4 and NANOG: targets identified solely by ChIP-Chip, ones identified only by ChIP-PET and another set uncovered using both methods. PCR was performed on the Ligation-mediated PCR products for two independent biological replicates for each protein. 10 ng of immuno-enriched DNA and 10, 30 and 90 ng of whole-cell extract DNA were used per reaction. The PCR was run for 23 cycles, and products were quantified on an agarose gel stained with SYBR Gold (Invitrogen). Primers for each target amplified a 200-300 bp region around the genomic locations of the probes showing enrichment. The primer sequences and PCR product coordinates can be found in [see Additional data file 13]. Enrichment was calculated as a ratio of intensity of PCR product from 10 ng of IP DNA to the product intensity from 90ng or 30 ng of WCE DNA. For a product to be considered enriched, the IP DNA product intensity had to be atleast that of 90 ng of WCE DNA or 1.5 times that of 30 ng of WCE DNA. Enrichment ratios were normalized against the ratio for un-enriched *β-Actin* DNA. Among NANOG bound targets found exclusively by ChIP-chip, 26 of 33 regions were confirmed by gene-specific PCR experiments. Similarly, 28 out of 31 OCT4-bound regions, identified solely by ChIP-chip were unenriched (**Table S9)**.

#### Gene expression analysis note

A caveat in this analysis was that the gene-expression comparison to binding data was limited to –4 to +4 kb surrounding the transcriptional start site for both ChIP-Chip and ChIP-PET data. Such a restriction was necessary for this comparison since probes on the promoter arrays used in ChIP-chip experiments are limited to these regions. Therefore, a binding event outside of this 8kb region in the ChIP-PET data would not be captured in our analysis. An example of this is demonstrated in [see Additional data file 3], where the *Rest* gene, which is differentially expressed on *Oct4* knockdown, is bound by OCT4 in both experiments. However, in our analysis of the expression data, it is observed as an OCT4 target only by ChIP-chip and not by ChIP-PET since the binding event in the latter case is outside the promoter array tiled region. Visualization of the two experimental types on the ‘GSE Visualizer’ reveals the utility of careful calibration of the thresholds at which binding events are called significant.

## References

1. LA Boyer, K Plath, J Zeitlinger, T Brambrink, LA Medeiros, TI Lee, SS Levine, M Wernig, A Tajonar, MK Ray, Bell GW, Otte AP, Vidal M, Gifford DK, Young RA, Jaenisch R: **Polycomb complexes repress developmental regulators in murine embryonic stem cells**. *Nature* 2006, **441**:349-53.

2. LA Boyer, TI Lee, MF Cole, SE Johnstone, SS Levine, JP Zucker, MG Guenther, RM Kumar, HL Murray, RG Jenner, Jenner RG, Gifford DK, Melton DA, Jaenisch R, Young RA: **Core transcriptional regulatory circuitry in human embryonic stem cells**. *Cell* 2005, **122**:947-56.

3. A Marson, K Kretschmer, GM Frampton, ES Jacobsen, JK Polansky, KD MacIsaac, SS Levine, E Fraenkel, H von Boehmer, RA Young: **Foxp3 occupancy and regulation of key target genes during T-cell stimulation**. *Nature* 2007, **445**:931-5.
